# RNA Virus Reassortment: An Evolutionary Mechanism for Host Jumps and Immune Evasion

**DOI:** 10.1371/journal.ppat.1004902

**Published:** 2015-07-09

**Authors:** Dhanasekaran Vijaykrishna, Reshmi Mukerji, Gavin J. D. Smith

**Affiliations:** 1 Duke-NUS Graduate Medical School, Singapore; 2 Yong Loo Lin School of Medicine, National University of Singapore, Singapore; 3 Department of Pathology, Singapore General Hospital, SingHealth, Singapore; 4 Duke Global Health Institute, Duke University, Durham, North Carolina, United States of America; University of Kentucky, Lexington, UNITED STATES

## Introduction

Reassortment is an evolutionary mechanism of segmented RNA viruses that plays an important but ill-defined role in virus emergence and interspecies transmission. Recent experimental studies have greatly enhanced our understanding of the cellular mechanisms of reassortment within a host cell. Our purpose here is to offer a brief introduction on the role of reassortment in segmented RNA virus evolution, explain the host cellular mechanisms of reassortment, and provide a synthesis of recent experimental findings and methodological developments. While we focus our discussion on influenza viruses, wherein most of the studies on reassortment have been carried out, the concepts presented are broadly applicable to other multipartite genomes.

## What Is Virus Reassortment?

**Fig 1:**
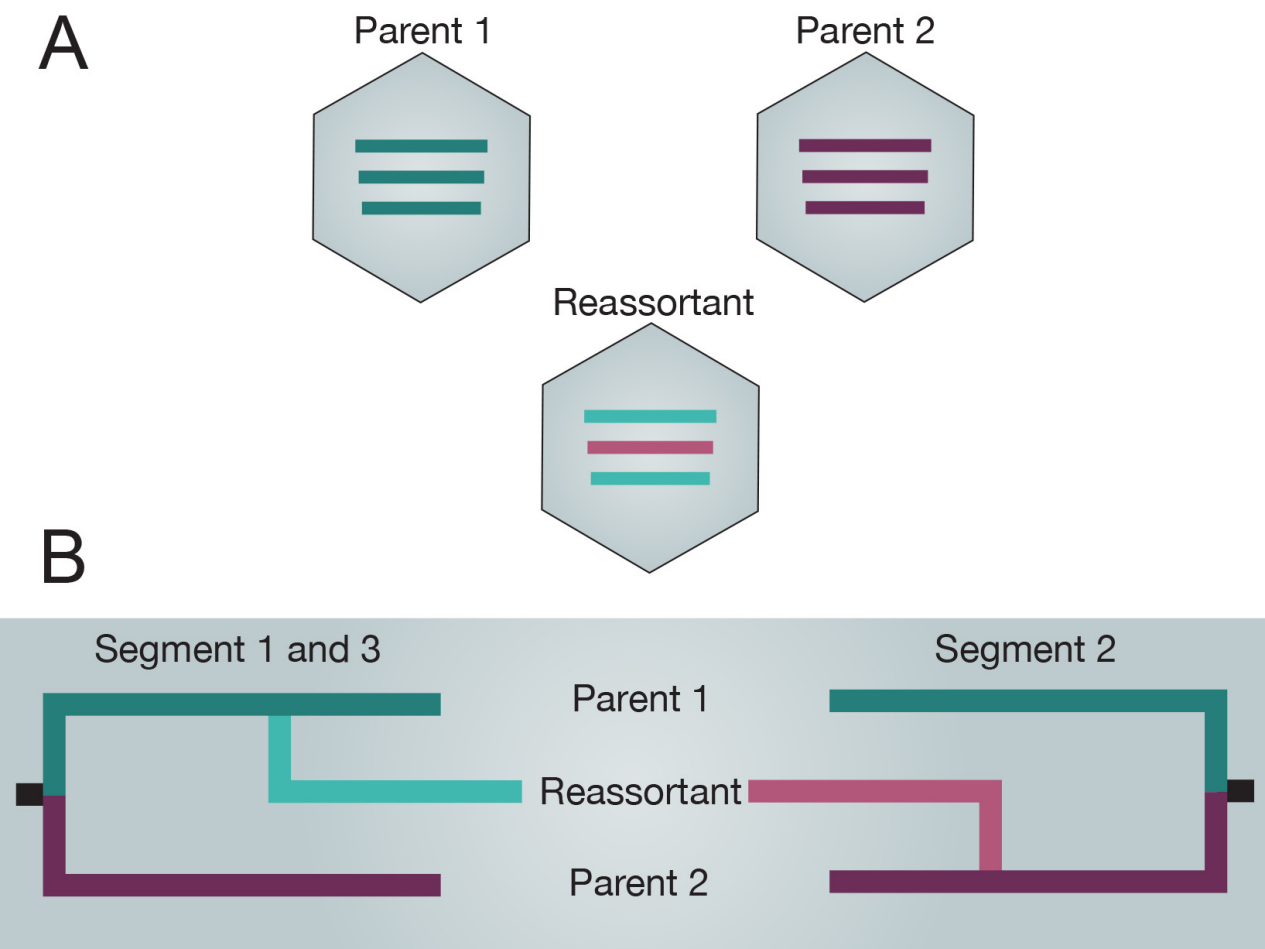
Reassortment of two tripartite genomes producing a novel reassortant. A) Diagrammatic representation of the emergence of a novel reassortant strain with genes derived from two parents. B) Phylogenetic discordance between segments 1 and 3 (left) and segment 2 (right) for three tripartite strains. Branches in bolder colors represent parental strains, whereas lighter colors represent the acquisition of gene segments from different parents to form a novel reassortant strain.

Virus reassortment, or simply reassortment, is a process of genetic recombination that is exclusive to segmented RNA viruses in which co-infection of a host cell with multiple viruses may result in the shuffling of gene segments to generate progeny viruses with novel genome combinations (Fig 1A) [1]. Reassortment has been observed in members of all segmented virus families, including, for example, Bluetongue virus [2], but reassortment is most prominently described for influenza viruses as a primary mechanism for interspecies transmission and the emergence of pandemic virus strains [3–5]. For instance, reassortment accelerates the rate of acquisition of genetic markers that overcome adaptive host barriers faster than the slower process of incremental increase due to mutation alone. The emergence of new influenza genes in humans and their subsequent establishment to cause pandemics have been consistently linked with reassortment of novel and previously circulating viruses [4–6].

In contrast, recombination occurs through a template switch mechanism, also known as copy choice recombination. When two viruses co-infect a single cell, the replicating viral RNA-dependant-RNA-polymerase can disassociate from the first genome and continue replication by binding to and using a second distinct genome as the replication template, resulting in the generation of novel mosaic-like genomes with regions from different sources [7,8] such as some circulating recombinant forms of HIV [9]. Although, in principle, recombination can occur in both segmented and non-segmented viruses, reports of recombination in segmented viruses have been frequently disputed [10,11] as weak evidence that arose through laboratory or bioinformatic artifacts [12,13]. Here we focus on virus reassortment using the well-studied influenza virus as an example.

## How Do Segmented Viruses Reassort within a Host Cell?

Essential prerequisites for reassortant include the entry of more than one virus particle into a single host cell, followed by the concomitant production of genome segments within the host cell. Experimental systems have revealed a high frequency of multiple infections [1,14], although there is some evidence suggesting the role of specific viral proteins limiting further infection [15].

Ultimately, the definitive formation of viable infectious reassortants is dependent on the incorporation of one copy of each segment into a virus particle. Two alternative mechanisms for reassortment within the host cell have been proposed. The random packaging model [16,17] posits that viral RNA is incorporated in virions without discrimination (but not other viral or cellular RNA); hence, the likelihood of forming viable reassortants with an entire genome set occurs by chance [16]. However, mounting evidence supports an alternative selective packaging model [18–20], which proposes that a virus particle packages eight unique viral RNA segments through specific packaging signals. Experimental visualization of RNA interactions [18] during virus assembly has revealed detailed interactive networks—i.e., epistatic interaction of virus packaging signals—among virus segments, which are thought to play an important role in directing reassortment. Through the experimental swapping of packaging signals between influenza viruses of different types, Essere et al. [19] were able to overcome the bias observed towards specific genotypes. In an extreme case, Baker et al. [19,21] have shown that the swapping of packaging signals of two different species of influenza viruses enabled reassortment to form viable particles that have not been observed in nature, indicating a central role for these packaging signals in reassortment. Intuitively, the emergence of differences in the packaging signals of diverging virus lineages may lead to virus speciation. Such a phenomenon could explain the lack of reassortment between the two influenza virus species (A and B) that share structural and functional similarities and that occupy the same ecological niche. Despite a lack of a mechanistic understanding of the function of packaging signals, these observational studies highlight important implications for viral evolution through epistatic interaction between gene segments and the emergence of novel reassortants.

## How Is Reassortment Detected?

The identification of reassortment is important to detect novel reassortants with increased transmissibility, increased pathogenicity, or those that escape antibody recognition or are resistant to antivirals. Reassortment is most commonly detected through incongruencies in phylogenetic relationships among the different segments of a viral genome [22–26], as gene segments from the same virus isolate occupy conflicting phylogenetic positions due to differences in their evolutionary histories (Fig 1B). Early studies identified reassortment by manually detecting phylogenetic incongruence of different viral segments. However, this method becomes impractical for studying large datasets, especially those with complex reassortment histories with nested reassortments or when there is a lack of phylogenetic support for reassortment among closely related sequences [27]. This has led to the development of several automated reassortment detection methodologies [28–31], but the phylogeny-based methods have remained the most robust and popular method for detecting reassortment [29,30]. Several extensions of the phylogenetic method have also been successfully applied to estimate past reassortment of viral lineages, including the coalescent-based Bayesian phylogenetics that infer and compare the time of most recent common ancestor (TMRCA) of each segment to infer possible reassortment [32], multi-dimensional scaling of tree distances [25,32], and more recently, using time-resolved Bayesian phylogenetics and trait state changes [33–35]. In addition, several distance-based methods exist [27], where degrees of similarity between pairs of viral genomes are used to infer reassortment [36,37]. Recently, a study has used a novel method based on the rapid rate of amino acid replacement post reassortment as a method of detecting a reassortment event [27]. While all the studies listed above are aimed at identifying reassortment events and strains, methodologies that infer an explicit rate of reassortment are rare, but examples include [33,34,38].

## What Do Genomic Studies Tell Us about Reassortment?

Influenza exhibits high levels of mixed infections in all major hosts [39–42], with up to 25% of all infections in avian hosts involving multiple influenza subtypes. However, large-scale genomic studies have identified various levels of restrictions on random reassortment between co-circulating influenza viruses, which differ depending on host, subtype, and preferential genetic combinations [35,36,43–46]. The greatest frequency of influenza reassortment is observed in their natural reservoir, wild aquatic birds [40], where viruses of different subtypes frequently exchange gene segments. However, reassortment is more restrictive in other hosts, particularly humans. Reassortment between human seasonal influenza viruses of different subtypes (A/H1 and A/H3 viruses) is rare [47] despite co-circulation over 40 years and extensive evidence of mixed infection [39]. Furthermore, studies of human influenza viruses have shown that certain combinations of gene segments were consistently detected in surveillance, suggesting either preferential assortment of these gene segments or a fitness advantage to these combinations. Convincing evidence comes from the two co-circulating and frequently reassorting lineages of influenza B viruses [35,48], but virions consistently contained the polymerase basic 1, 2, and the hemagglutinin (HA) genes (PB1-PB2-HA) from a single lineage [35]. Similarly, preferential combinations of segments are transiently observed for human influenza A viruses [45,46].

## What Are the Consequences of Virus Reassortment?

The tremendous genomic novelty generated by reassortment confounds all current methods of virus control. Evolutionary studies indicate an advantage for gene lineages with reassorting backgrounds. Specifically, a significant increase in transient amino acid mutations is observed following reassortment [27], primarily in the surface glycoprotein hemagglutinin, the major immunogenic protein of influenza that leads to antigenic change [25,32]. This suggests that the placement of the HA within novel genetic backgrounds through reassortment greatly affects virus fitness and directly influences antigenic variation, contributing to the long-term evolution of the virus. However, reassortment could lead to evolutionary change due to various other factors, including selection pressure induced by herd immunity; the residues being under weak selective constraint; or compensation for fitness costs of mutations accruing elsewhere in the genome [25]. Similarly, the emergence of drug-resistant mutations may be acquired following reassortment, as shown for the emergence of amantadine-resistant H3N2 viruses [49] and oseltamivir-resistant seasonal H1N1 viruses [50]. These studies suggest that reassortment confounds available methods of virus control, although detailed examination of the role of reassortment in driving genome-wide evolution is still needed.
